# The Importance of Heat Control During Dental Implant Positioning: An In Vitro Thermal Analysis

**DOI:** 10.1155/bmri/7910355

**Published:** 2026-03-10

**Authors:** Dario Milone, Davide Crisafulli, Dario Santonocito, Luca Fiorillo

**Affiliations:** ^1^ Department of Engineering, University of Messina, Messina, Italy, unime.it; ^2^ Department of Dental Research Cell, Dr. D. Y. Patil Dental College & Hospital, Dr. D. Y. Patil Vidyapeeth (Deemed to Be University), Pimpri, Pune, India; ^3^ Department of Medicine and Surgery, University of Enna “Kore”, Enna, Italy

**Keywords:** dental implant procedures, implant site preparation, osseointegration, thermal analysis, thermographic cameras

## Abstract

The use of thermal analysis in dental implant procedures marks a significant advancement, allowing the study of temperature changes during implant placement. Research has demonstrated the value of thermal imaging in assessing tissue health, detecting early damage, and understanding biomechanical interactions during these procedures. The study involved testing the heat generated when screwing titanium dental implants (Diagram, Schutz‐Dental GmbH, Rosbach, Germany) into resin D3 bone‐like blocks that mimic bone′s mechanical properties. Various screwing protocols were evaluated, considering the presence or absence of lubricant, the type of instrument used (manual or micromotor), and the number of revolutions. Temperature changes on the surface of the implant were monitored with a microbolometric infrared camera, FLIR A40. In total, 24 implants were placed using different protocols. Results indicated that the highest temperature rises occurred during manual screwing without physiological solution, especially in undersized preparation sites where friction was increased. Adding lubrication significantly lowered the surface temperature, although water buildup affected measurement accuracy. Overall, the findings showed that screwing speed, lubrication, and preparation size influence thermal behavior during implant placement. The study concludes that thermal analysis is essential for understanding dental implant procedures, with thermocameras providing a noninvasive method for examining thermal dynamics during insertion. This can lead to improved implant designs and techniques that reduce thermal damage to surrounding tissues, thereby enhancing success rates and safety. The research emphasizes the need for further in vivo studies, the development of new materials for drills and implants, and personalized drilling protocols based on patient‐specific factors. It also highlights the importance of education and training on thermal analysis for dental professionals and considers regulatory and ethical issues in advancing these technologies.

## 1. Introduction

Thermal analysis represents an innovative methodology for examining temperature variations during dental implant insertion [1]. This technique facilitates safe and efficient implant placement, consequently mitigating the risk of complications. Thermal analysis aids in identifying the optimal implant position to achieve superior clinical outcomes [2, 3]. So, to experience a successful dental implant procedure, ensure that your dentist uses thermal analysis during implant positioning [1, 2, 4–6]. The application of thermographic cameras in bioengineering signifies a significant advancement in the field. By detecting infrared radiation, these cameras offer a noninvasive, detailed methodology for observing and analyzing various biological processes and structures. For example, thermal imaging has been utilized to evaluate skin health, as demonstrated in the study by Handley and Hessefort [7], in which the skin condition of healthcare workers′ hands was analyzed over a year. In dental bioengineering, Matos et al. [8] highlighted the application of thermographic cameras alongside other tools for in vitro and silico analyses, proving its importance in modern dentistry.

Moreover, Zhao et al. [9] demonstrated the effectiveness of thermographic cameras in detecting cracks in frozen soils, showcasing the camera′s versatility beyond traditional bioengineering applications. Hoshino et al. [10] investigated the use of drones with thermographic cameras for disaster victim detection. In medical diagnostics, Vasconcellos et al. [11] effectively used thermographic cameras to assess small fiber peripheral neuropathy in prediabetes, highlighting their potential for early diagnosis. These diverse applications underscore the critical role of thermographic cameras in bioengineering and related disciplines, providing innovative methodologies for research and diagnosis [2]. The utilization of thermographic cameras to evaluate thermal changes during dental implant procedures constitutes an innovative approach within dental bioengineering. These devices uniquely facilitate the monitoring and recording of temperature variations, offering valuable insights into the thermal effects occurring during implant insertion. In vitro, a thermocamera can be employed to analyze how factors such as implant material, design, insertion speed, and bone density influence the thermal environment during placement. In vitro, a thermocamera can analyze how factors such as implant material, design, insertion speed, and bone density influence the thermal environment during implant placement. For example, studies such as those by Demirbaş et al. [12] demonstrate this approach. They [12] have explored the distributions of stress and damage during dental implant insertion, highlighting the importance of understanding the biomechanical interactions involved in such procedures. Similarly, Yang et al. [13] developed an analytical model for dental implant insertion torque, considering factors that could influence thermal changes during implant placement. Moreover, research by Klär et al. [14] combined strain gauge and histologic analysis to study bone damage during dental implant insertion, which can be complemented by thermographic analysis to understand the thermal impact on bone tissue. Varghai et al. [15] investigated the effect of drilling speed on dental implant insertion torque, which can significantly influence thermal generation and can be effectively monitored using thermocameras. These studies highlight the potential of thermocameras as a noninvasive and accurate method for assessing thermal behavior during dental implant insertion. This methodology holds the potential to facilitate the development of enhanced implant designs and insertion techniques capable of mitigating thermal injury to adjacent tissues, consequently augmenting the overall success and safety of dental implant procedures.

## 2. Materials and Methods

### 2.1. The Test

The following section describes the experimental approach adopted to evaluate heat generation during the insertion of dental implants into resin blocks. These blocks were chosen for their ability to mimic the mechanical properties and consistency of D3 type bone, providing a reliable model for in vitro experimentation and implant surgical practice.

### 2.2. Experimental Setup and Sample Preparation

The experiment was conducted under strictly controlled environmental conditions to ensure the reproducibility of the results. The test assesses the heat generated when dental implants are screwed into resin blocks with D3 bone consistency, used for practicing implant surgery and mimicking the mechanical properties of bone. These blocks measure 2 × 2 × 5 cm initially. Using the specialized diagram surgical kit, 4‐mm diameter and 10‐mm deep preparations were made in the resin to ensure proper implant placement. A single 4 by 10 mm implant (Diagram, Schutz‐Dental GmbH, Rosbach, Germany) was inserted into a 3.5‐mm preparation to examine how underpreparation influences temperature rise [16]. The details are shown in Table 1.

**Table 1:** Experimental setup and sample preparation details.

Parameter	Specifications
Sample material	Resin blocks (D3 bone analog)
Initial dimensions	2 × 2 × 5cm
Ambient temperature	23°C
Relative humidity (RH)	50%
Surgical kit used	Diagram (Schutz‐Dental GmbH)
Implant used	4‐mm diameter, 10‐mm length
Preparation diameter	3.5 mm

### 2.3. Thermal Measurement Methodology

An infrared thermal camera was used to measure the increase in surface temperature during implant insertion. To optimize the accuracy of infrared radiation detection and eliminate interference caused by the intrinsic low thermal transmittance of the resin, the emissivity of the external surface was standardized. Each block was coated with black matte paint, bringing the surface emissivity to a known and constant value:

• Surface treatment: black matte paint
• Emissivity value (*ε*): 0.98

The recorded measurement was not the absolute temperature, but the variation in temperature (*Δ* *T*) from the test starting point, allowing for a precise analysis of the thermal energy dissipated during the procedure. The technical specifications of the thermal camera used are summarized in Table 2.

**Table 2:** Infrared camera specification.

Thermal sensitivity	0.08°C at 30°C (@ 50/60 Hz)
Spatial resolution (IFOV)	1.3 mrad
Spectral range	7.5–13 *μ*m
Detector type	Focal plane array
Temperature range of measurement	Range 1: −40°C to +120°C (−40°F to +248°F)
Accuracy (% of reading)	± 2°C or ± 2%

### 2.4. Experimental Protocols and Thermal Analysis

Various implant insertion protocols, each with specific characteristics, were assessed to understand their thermal impact at the bone–implant interface. The temperature data, captured by the infrared thermal camera, specifically measured the localized heat generated at the interface during the active phase of implant screwing under both dry and wet conditions.

### 2.5. Tested Variables

The study systematically evaluated various procedural variables that can influence the outcome of dental implant insertion, with a particular focus on thermal and mechanical impact. The objective was to compare the performance and average temperature values generated under specific protocols. The variables systematically evaluated for implant insertion were as follows:

• Cooling/lubrication conditions:
  o. Wet condition (with physiological solution): In this scenario, the physiological solution was used as a lubricant to mimic the presence of bodily fluids (blood and saliva) at the surgical site, rather than as an active cooling agent. This approach mimics a realistic clinical environment, where biological fluids contribute to heat management and natural lubrication.
  o. Dry condition: Insertion performed without the application of any solution, a control condition likely to generate greater friction and subsequent temperature increase.
• Instrument used for implant placement:
  o. Surgical micromotor: Used with a maximum insertion torque limited to 50 Ncm. This is a common and standardized method in implant surgery, as torque values above 50 Ncm can lead to greater peri‐implant bone resorption.
  o. Manual torque ratchet: Used for manual insertion, with a maximum applicable torque of up to 100 Ncm. Manual use allows the surgeon to “feel” the bone resistance and apply potentially higher torque, which may be indicative of greater primary stability.
• Number of revolutions (revolutions per minute):
  o. Several standardized speeds were tested for mechanical insertion: 30, 50, 75, and 100 RPM. Previous studies have shown that lower speeds (like 30 or 50 RPM) generate less heat than higher speeds (like 100 RPM), reducing the risk of bone necrosis.
  o. The manual option (with a variable number of revolutions) provided a clinical comparison point with the mechanized approach.

Before proceeding with the measurements, the specimens were securely clamped in a bench vice to ensure stability and prevent any unwanted movement during the implant insertion phase.

For each of the different protocol combinations (wet/dry condition, instrument type, and number of revolutions), two implants were tested. The average temperature values recorded during these insertions were then calculated and compared to determine the relative impact of each variable.

This rigorous methodology allowed for isolating and quantifying the specific effect of each parameter on the conditions of the implant site, a crucial aspect for defining optimal clinical protocols and preventing thermal damage to the surrounding bone.

## 3. Results

The results from combining all different protocols showed that an expert operator placed 24 dental implants, either manually or with a surgical micromotor, following 12 protocols created by combining various options from the previous section. The data, summarized in Table 3 and explained in detail later, include values labeled with IDs 1 and 2, representing dry and nondry manual implant positioning. Subsequent protocols (ID3 and ID4) involved 30 RPM dry and nondry procedures, progressing to 100 RPM dry and nondry protocols at ID9 and ID10. The final two protocols (ID11 and ID12) assessed borderline conditions often used in clinical practice, such as underpreparation or high‐torque implant placement. These involved inserting the implant at 30 RPM and completing preparation with a dynamometric ratchet until reaching 100 Ncm, without lubricant, or underpreparing the implant site by 0.5 mm. Generally, higher screwing speeds resulted in higher maximum surface temperatures. Lubrication with water reduced surface temperature, as shown in Figure 1, which plots temperature over time. The left *y*‐axis shows absolute temperature at the hole′s edge, while the right *y*‐axis indicates the temperature change from start to end. The temperature increase (delta) was 0.4°C for lubricated sites and 4.1°C for dry sites. However, during screwing, water accumulates around the hole, complicating temperature measurement (see Figure 2), as surface water affects both the accuracy of temperature readings and the magnitude of temperature variation.

**Table 3:** Mean data about the different protocols.

ID	Speed (RPM)	*T*_*i*_ (°C)	*T*_*f*_ (°C)	*Δ* *T*	Notes
1	Manually	25.2	25.6	0.4	Water
2	Manually	30.1	34.2	4.1	No cooling
3	30	24.7	25.6	0.9	Water
4	30	27.4	28.9	1.5	No cooling
5	50	27.2	28.5	1.3	Water
6	50	26.8	28.9	2.1	No cooling
7	75	27.6	29	1.4	Water
8	75	29.1	31	1.9	No cooling
9	100	25.6	28.5	2.9	Water
10	100	29	32.4	3.17	No cooling
11	30+ manually	29.2	40.2	11	No cooling, smaller preparation (3.5 mm)
12	30+ manually	27.4	33.1	5.7	No cooling

Figure 1Temperature trend during manual screwing Test ID 01 and 02: (a) with water and (b) no cooling.

**Figure:**
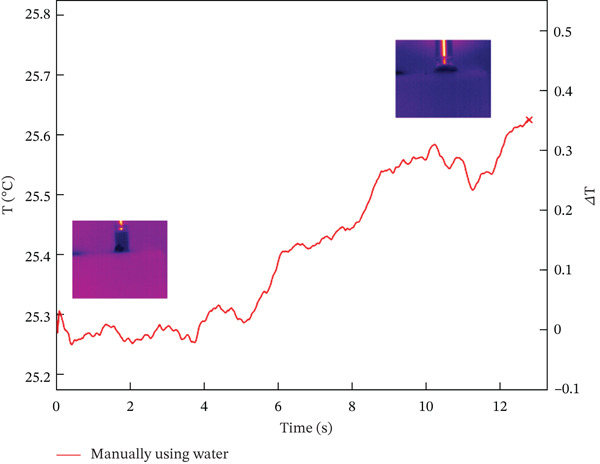
(a)

**Figure:**
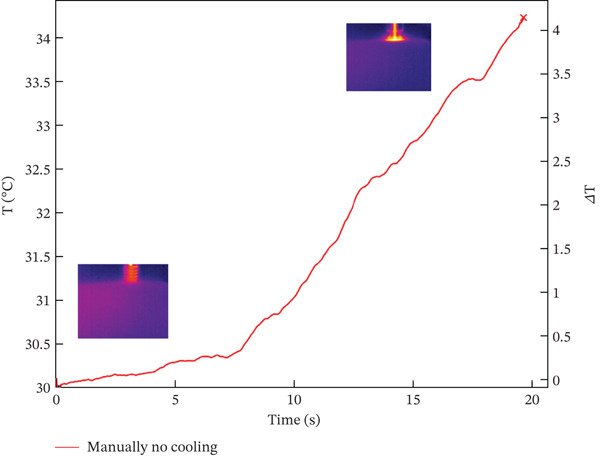
(b)

Figure 2ID screwing Test 03 with water lubrification: (a) before screwing and (b) after screwing.

**Figure:**
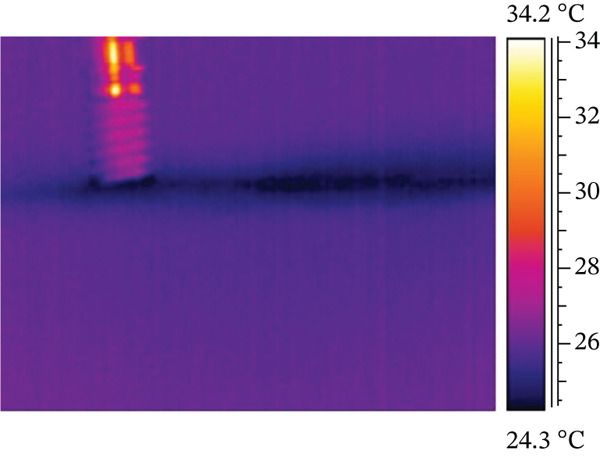
(a)

**Figure:**
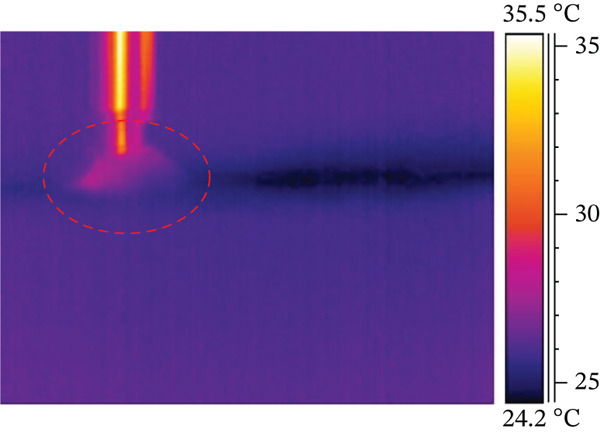
(b)

**Figure 4:**
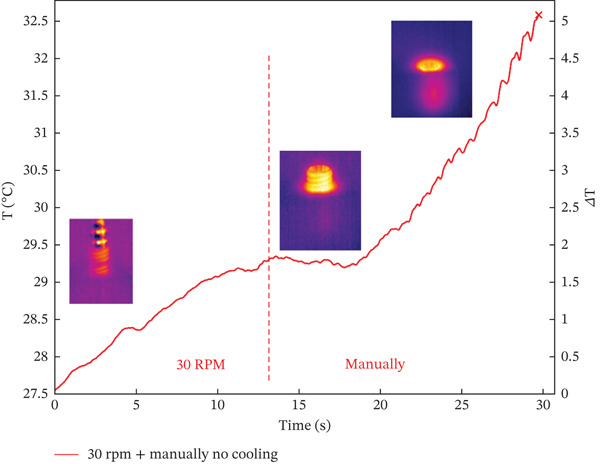
Temperature trend during Test ID 12, 30 RPM + manually.

A smaller preparation site reaches the highest temperature difference in the test, possibly due to friction between the titanium implant and the resin base during screwing. Both electric motor and manual screwing methods were tested, with manual screwing showing the most significant temperature increase. Figure 3 illustrates the three stages of the combined screwing process: Figure 3a shows the initial stage before starting, Figure 3b depicts the temperature distribution at the end of motorized screwing and before manual screwing, and Figure 3c displays the heat map after manual screwing. Figure 4 illustrates temperature changes over time for both methods, with a plateau in the middle indicating the point at which the operator switched methods.

Figure 3ID screwing Test 12 with no cooling: (a) before screwing, (b) after micromotor screwing, and (c) after manual screwing.

**Figure:**
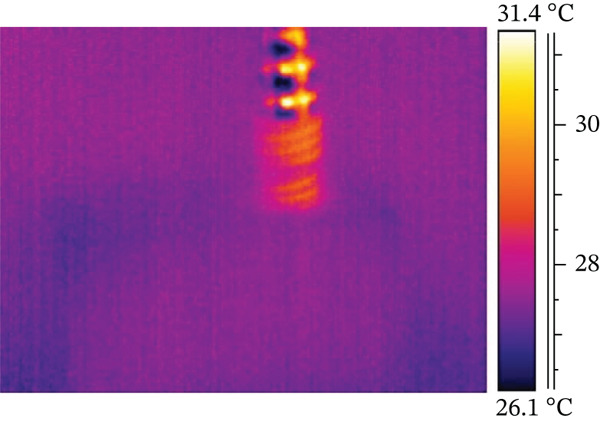
(a)

**Figure:**
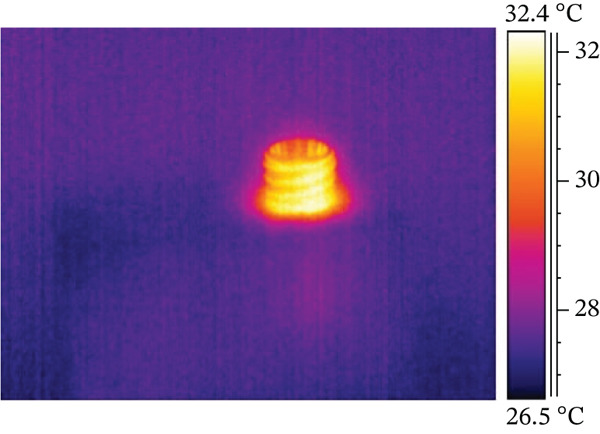
(b)

**Figure:**
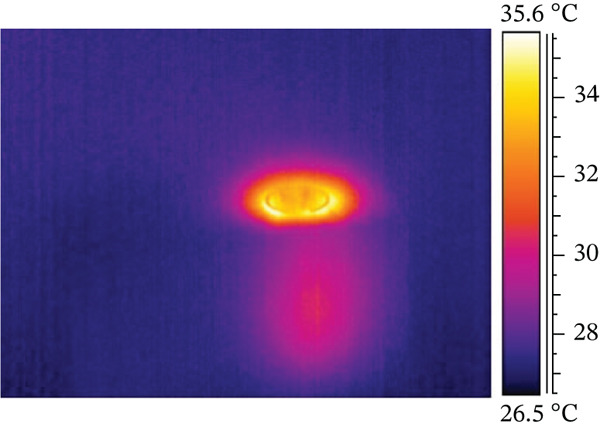
(c)

## 4. Discussion

The crucial limit during bone surgery is avoiding thermal osteonecrosis, which is the death of bone tissue caused by excessive temperature. Osteonecrosis occurs when the blood supply to a bone is disrupted, resulting in damage to the bone and cartilage and impairing joint function. It can also harm prosthetic implants and promote the growth of infections. Maintaining temperatures below 47°C for over a minute is crucial, as it is vital to minimize heat and mechanical stress during implant drilling. Research has extensively examined heat creation during drilling, aimed at finding effective ways to prevent overheating. Deeper drillings increase heat, especially with traditional and piezosurgical drills. For example, a 13‐mm depth with a piezoelectric drill reached 38.1°C, while conventional drills peaked at 33.6°C, both of which are below the critical threshold. Studies comparing guided and conventional surgeries indicate that guided procedures, especially those using larger drills (2.2, 3.5, and 4.2 mm), present a higher risk of tissue necrosis because the surgical guide hampers the flow of cooling water and increases friction, resulting in increased heat. Repeated drilling, rather than single‐pass, further raises the risk of overheating at the implant site [17, 18].

Variations in irrigation volume and the load applied to the drill were also examined. Conventional, sonic, and ultrasonic drills were used on both cortical and spongy bone layers. Increasing the irrigation volume led to lower temperatures during all tested osteotomies. Conventional rotary instruments caused a temperature rise of 6.5°C at an irrigation rate of 20 mL/min, which decreased with higher irrigation volumes. However, no tests reached the critical temperature of 47°C [19].

The material and design of the drill, as well as its frequency of use, also impact bone temperature during site preparation under consistent conditions of a 20‐kg load and a 1500 RPM speed. Steel drill bits produced less heat than ceramic ones. Maximum average temperatures were recorded at depths of 3, 6, and 9 mm, with ceramic drills initially generating higher temperatures, likely due to their lower thermal conductivity. However, the temperature never reached the critical threshold of 47°C during the tests [20]. A review by Dhok et al. [17] comprehensively analyses the thermal dynamics in dental implant procedures, emphasizing the relationship between heat generation and successful osseointegration. It highlights that temperature rise during implant operations can lead to thermal injury of the adjacent bone tissue, potentially causing implant failure. The paper thoroughly examines factors such as drill speed, design, irrigation mode, depth, diameter, and drill material, and how they influence osseointegration due to heat generated during drilling—finite element analysis (FEA) studies, including those by Ahmadi and Mohammadi [21], have been utilized to assess the impact of drilling parameters on heat generation. These studies demonstrate that higher feed rates result in smaller temperature increases and that water application significantly reduces maximum temperatures. The implant diameter is a crucial design factor, with research indicating that guided osteotomy preparation and sequential drilling protocols can lead to greater temperature deviations compared to traditional methods. Drill speed is a crucial factor in dental implant procedures, with research showing that higher speeds, such as 2500 RPM, produce less heat and shorten preparation time, while slower speeds generate more frictional heat. However, very low speeds can cause the drill to become stuck, increasing heat production. The depth of drilling, determined by the bone thickness where the implant is placed, also significantly influences heat generation, with deeper drilling leading to higher temperatures. Irrigation is essential for effective osteotomy, as it reduces friction, aids efficient drilling, cools the drill bit and surrounding tissues, and helps prevent infection. The effectiveness of irrigation in managing rising temperatures is well established, with external irrigation being more effective than internal methods.

Furthermore, the amount of irrigation during site preparation sometimes results in significantly different temperature controls compared to a moderate volume. The review emphasizes the importance of considering factors such as drill speed, diameter, depth, and irrigation to prevent heat‐induced implant failure. Keeping the temperature within specific limits is essential to avoid thermal necrosis. Selecting appropriate drill parameters and irrigation methods is crucial for managing temperature increases during dental implantation. The review also calls for further research to identify optimal parameters for effective implant site preparation and postoperative care. This comprehensive analysis underscores the need to control heat generation factors to ensure successful and safe dental implants. Tur et al. conducted a study examining the thermal performance of metal (stainless steel) and ceramic (zirconia) implant drills, focusing on temperature exposure time during osteotomy. The study involved 240 procedures, considering variables like drilling depths (10 and 16 mm), irrigation methods (external and none), drill materials, and three drill diameters per material (2.0/2.2, 2.8, and 3.5 mm), with 10 repetitions each. Real‐time temperature measurements were taken during automated drilling in standardized bovine bone specimens. Results showed that the highest temperature changes were mainly linked to the drill withdrawal phase, regardless of drill material, depth, or diameter. Statistically significant differences in temperature rise were found between stainless steel and ceramic drills in irrigated testing sites, especially with smaller drill sizes (2.0/2.2 and 2.8 mm). The study revealed a strong link between the highest temperature increases and the passive withdrawal period for both drill types. These findings indicate that elevated temperatures, along with extended heat exposure during surgery, could affect the osseointegration process over time. Additionally, other research has examined changes in implant surface temperature after implantoplasty to address peri‐implantitis. Sharon et al. [22] explored the efficiency of different dental burs in implantoplasty, a treatment option for peri‐implantitis, and the amount of heat generated by each bur. The study assessed the effectiveness of three types of dental burs—diamond, diamond from the Premium Line, and carbide—in removing titanium implant material, comparing them with a smooth bur used as a control. Each bur was connected to a high‐speed handpiece and applied to a titanium implant for 60 s, with water spray cooling. Temperature changes were recorded every 5 s, and the amount of material removed was measured by the reduction in implant weight. Results revealed notable differences: the diamond Premium Line bur removed 59.24 mg, the carbide bur 29.39 mg, the standard diamond bur 11.35 mg, and the smooth bur only 0.19 mg. Thermally, only small increases of about 1.5°C were observed across all burs. The findings indicate significant variability in burs′ efficiency when working on titanium, suggesting that choosing the right bur can save time. Proper cooling during implantoplasty prevents excessive temperature rise that could harm surrounding soft tissue or bone. The deltas across all protocols should be evaluated to achieve acceptable values both without lubrication and in the presence of simulated blood and saliva (using a physiological solution). Specific data analysis reveals significant findings regarding cooling and speed. Across several tests, the average temperature increases *Δ* *T* was 2.86°C but varied widely from 0.4°C to 11.0°C. A critical observation is the substantial difference based on cooling method. Tests without water cooling resulted in an average *Δ* *T* of 4.73°C, nearly four times higher than tests with water cooling (average *Δ* *T* of 1.20°C). Furthermore, while higher revolutions per minute generally increase temperature, the effectiveness of water cooling decreased as speed increased (the *Δ* *T* difference between cooled and noncooled tests diminished from 3.7°C at manual speeds to only 0.27°C at 100 RPM). The only exceptions are the values obtained in experimental protocol ID11, where the implant site is underprepared. Therefore, considering the temperature of the environments where the studies were carried out as the baseline, the maximum temperature reached was 40.2°C, with a delta of 11°C. However, considering the body temperature of 37°C as the starting temperature and not accounting for the state transitions of the tested material [23], temperatures of up to 48°C could occur with the ID11 protocol, which could lead to osteonecrosis in this scenario. Meanwhile, in the ID10 protocol, the temperature rises from 37°C to 40.17°C during implant fixture screwing at 100 RPM. Therefore, it is crucial to prevent both overheating and improper cooling of the bone after preparing the implant site. It is recommended to cool the site after removing the final drill from the surgical kit and then proceed with implant placement, using a low number of revolutions and a torque not exceeding 50 Ncm to prevent thermal osteonecrosis [22, 24, 25]. Another important aspect is the use of implant protocols with piezosurgery. Even when these instruments are used for preparation, the implant must still be placed by screwing. As a result, we encounter the same issue repeatedly [26]. This issue may be influenced by implants that lack threaded designs and platforms requiring precise positioning during percussion implant preparation. It is essential to assess the extent of osteonecrosis and the tissue′s capacity to promote healing and osteointegration of the implant screw. Additionally, biopsies and in vivo studies are necessary. Previous studies have shown that the design and material of the drill significantly affect thermal behavior during implant site preparation, impacting peak temperature, duration of heat exposure, and the biological response. Comparative thermographic and infrared analyses have shown that zirconia and steel drills behave differently under clinical irrigation conditions, while single‐drill protocols may generate distinct thermal profiles relative to traditional sequential drilling approaches. Moreover, studies assessing bur wear, drill efficiency, and osteotomy morphology have confirmed that instrument degradation and geometric changes can considerably alter frictional heat generation during bone cutting. Complementary computational and finite element investigations have further highlighted the mechanical and thermal consequences of different implant systems, insertion strategies, and material properties, supporting the need for integrative modeling when evaluating heat‐related risks. Finally, multidisciplinary research in materials science and orthodontic biomechanics reinforces the importance of microscopic, thermographic, and spectroscopic assessment to fully characterize temperature‐dependent phenomena at the bone–implant interface [27–33]. A primary limitation of this study is the use of a resin with the same consistency as D3 medullary bone. Furthermore, a more significant limitation is the current consideration to perform the same procedure on bovine bone specimens to better replicate real‐world mechanical behavior. Future development is aimed at involving clinical studies with human subjects in the operating room to obtain more veridical results. It is also important to note that this research did not consider situations where the implant itself was precooled; future studies should investigate whether variations in the initial implant temperature alter the thermal response curve.

## 5. Conclusion

The study highlights the innovative role of thermal analysis in dental implants. Using thermographic cameras helps practitioners position implants more safely and efficiently, reducing complications. This approach allows for optimized implant placement and better patient outcomes. Thermocameras are especially valuable in dental bioengineering, providing a noninvasive way to monitor temperature changes during implant procedures. They offer detailed thermal imaging, helping to understand how factors like material, design, insertion speed, and bone density influence the thermal environment. Future advances in dental implantology will focus on several areas. Greater research is needed to understand how thermal dynamics impact osteointegration and tissue healing; in vivo studies, including biopsies, will be essential. Ongoing research into drill and implant materials is aimed at developing options that reduce thermal effects, emphasizing materials with ideal thermal properties. Personalized drilling and insertion protocols, based on patient‐specific factors like bone density, could improve safety and success rates. Education for dental professionals must keep pace with technological advances, ensuring they are trained in new thermal analysis methods. Additionally, addressing regulatory and ethical issues will be vital to protect patient safety and privacy as these technologies develop.

## Author Contributions

Concept/design and supervision, L.F. and D.M.; data analysis/interpretation, data collection, and visualization, D.M., D.S., and D.C.; drafting article, L.F., D.M., and D.S.; critical revision of article, L.F.; and approval of article, L.F., D.M., D.S., and D.C.

## Funding

No funding was received for this manuscript. Open access publishing facilitated by Universita degli Studi di Messina, as part of the Wiley ‐ CRUI‐CARE agreement.

## Disclosure

This paper has been presented as a poster presentation at the FDI World Dental Congress [16], with no details about the definitive manuscript or data. All authors accepted and agreed on the final version of the manuscript.

## Conflicts of Interest

The authors declare no conflicts of interest.

## Data Availability

The data that support the findings of this study are available from the corresponding author upon reasonable request.
